# Enantiopure Chiral Coordination Polymers Based on Polynuclear Paddlewheel Helices and Arsenyl Tartrate

**DOI:** 10.3390/polym10030311

**Published:** 2018-03-13

**Authors:** Ángela Valentín-Pérez, Ahmad Naim, Elizabeth A. Hillard, Patrick Rosa, Miguel Cortijo

**Affiliations:** 1 Centre National de la Recherche Scientifique, Centre de Recherche Paul Pascal, UMR 5031, 33600 Pessac, France; valentin@crpp-bordeaux.cnrs.fr; 2 Université de Bordeaux, Centre de Recherche Paul Pascal, UMR 5031, 33600 Pessac, France; 3 Centre National de la Recherche Scientifique, Institut de Chimie de la Matière Condensée de Bordeaux, UMR 5026, 33600 Pessac, France; ahmadnaim@live.com (A.N.); patrick.rosa@icmcb.cnrs.fr (P.R.); 4 Université de Bordeaux, Institut de Chimie de la Matière Condensée de Bordeaux, UMR 5026, 33600 Pessac, France

**Keywords:** chiral coordination polymers, enantiomeric resolution, extended metal atom chains, circular dichroism, X-ray crystallography

## Abstract

Herein, we report the preparation of chiral, one-dimensional coordination polymers based on trinuclear paddlewheel helices [M_3_(dpa)_4_]^2+^ (M = Co(II) and Ni(II); dpa = the anion of 2,2′-dipyridylamine). Enantiomeric resolution of a racemic mixture of [M_3_(dpa)_4_]^2+^ complexes was achieved by chiral recognition of the respective enantiomer by [Δ-As_2_(tartrate)_2_]^2−^ or [Λ-As_2_(tartrate)_2_]^2−^ in *N*,*N*-dimethylformamide (DMF), affording crystalline coordination polymers formed from [(Δ-Co_3_(dpa)_4_)(Λ-As_2_(tartrate)_2_)]·3DMF (Δ-**1**), [(Λ-Co_3_(dpa)_4_)(Δ-As_2_(tartrate)_2_)]·3DMF (Λ-**1**), [(Δ-Ni_3_(dpa)_4_)(Λ-As_2_(tartrate)_2_)]·(4 − *n*)DMF∙*n*Et_2_O (Δ-**2**) or [(Λ-Ni_3_(dpa)_4_)(Δ-As_2_(tartrate)_2_)]·(4 − *n*)DMF∙*n*Et_2_O (Λ-**2**) repeating units. UV-visible circular dichroism spectra of the complexes in DMF solutions demonstrate the efficient isolation of optically active species. The helicoidal [M_3_(dpa)_4_]^2+^ units that were obtained display high stability towards racemization as shown by the absence of an evolution of the dichroic signals after several days at room temperature and only a small decrease of the signal after 3 h at 80 °C.

## 1. Introduction

The study of the self-assembly of molecular building blocks into polymeric structures extended in one, two, or three dimensions opens the way not only to a vast quantity of diverse materials but also to multiple applications. Specifically, chiral Coordination Polymers (CPs) offer a great potential in the context of numerous fields such as enantioselective catalysis [1,2,3,4], nonlinear optics [5,6], ferroelectricity [7], and magnetochiral dichroism [8]. Such enantiopure complexes can be obtained fortuitously by spontaneous resolution during crystallization [9,10] or by design, using chiral ligands, metalloligands [11,12,13], or chiral inductors, such as solvents, templates, or counteranions [14,15,16]. However, and despite the progress made in this direction, rational preparation of chiral CPs still presents a challenge in coordination chemistry.

We are here interested in the family of clusters known as “extended metal atom chains” or “metal strings”. These terms refer to polynuclear paddlewheel complexes with three or more linearly-arranged metal atoms bridged by four equatorial ligands, typically oligopyridylamines. These compounds, often containing metal-metal bonds, have been extensively studied since the late 1990s because of their fascinating magnetic and electronic properties [17,18,19]. Much less studied, however, is the use of such complexes to build extended structures, with only few one-dimensional (1D) and two-dimensional (2D) CPs based on [Co_3_(dpa)_4_]^2+^ [20,21,22] and [Ni_3_(dpa)_4_]^2+^ [23,24] (dpa = the anion of 2,2′−dipyridylamine) to be found in the literature.

**Figure 1:**
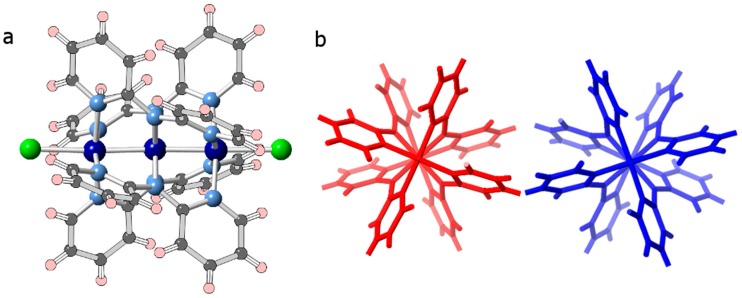
(**a**) Ball and stick representation of [Co_3_(dpa)_4_Cl_2_]. Cobalt: dark blue, nitrogen: light blue, chlorine: green, carbon: gray, hydrogen: pink; (**b**) Representation along the M−M−M axis of [Δ-Co_3_(dpa)_4_Cl_2_] (red) and [Λ-Co_3_(dpa)_4_Cl_2_] (blue). Figures generated from X-ray diffraction data.

Another relatively unexplored feature of such linear cluster complexes is their helicoidal chirality. This feature arises from the mutual steric hindrance of the 3-pyridyl protons, resulting in the twisting of the dpa ligands around the metal axis (Figure 1). Among the notable examples where enantiopure compounds have been obtained, we can cite the work by Cotton et al. in the chiral resolution of a racemic mixture of [Ni_3_(dpa)_4_Cl_2_] by chromatography using a macrocyclic glycopeptide-based chiral stationary phase [25,26]. This separation yielded enantiopure mixtures of [Ni_3_(dpa)_4_Cl_2_] and [Ni_3_(dpa)_4_Cl(OH)], which displayed enormous specific rotation values of ca. ±5000 deg∙mL∙g^−1^∙dm^−1^, comparable to those observed for some helicenes [27]. In another elegant work, Peng and coworkers were able to obtain enantiopure Ni_5_-based paddlewheel complexes using naphthyridylpyridyldiamine ligands functionalized with bulky chiral groups [28].

Recently, an approach based on anion exchange has been employed by our group to resolve a racemic mixture of [Co_3_(dpa)_4_(MeCN)_2_]^2+^ clusters [29]. For this purpose, inexpensive chiral dianions based on commercially available (2R,3R)-(+)- or (2S,3S)-(−)-tartaric acid were employed, affording the molecular species [Δ-Co_3_(dpa)_4_(MeCN)_2_](NBu_4_)_2_[Λ-As_2_(tartrate)_2_]_2_ and [Λ-Co_3_(dpa)_4_(MeCN)_2_](NBu_4_)_2_[Δ-As_2_(tartrate)_2_]_2_, respectively, by selective crystallization. Mirror-image Electronic Circular Dichroism (ECD) spectra of the enantiomers in acetonitrile were obtained, similar to those that were reported by Cotton et al. for the spontaneously resolved enantiomers of [Co_3_(dpa)_4_(MeCN)_2_][PF_6_]_2_ [30]. Furthermore, significant X-ray natural circular dichroism (XNCD) signals were also detected at the Co K-edge in circularly polarized X-ray absorption experiments made on oriented single crystals [29].

**Figure 2:**
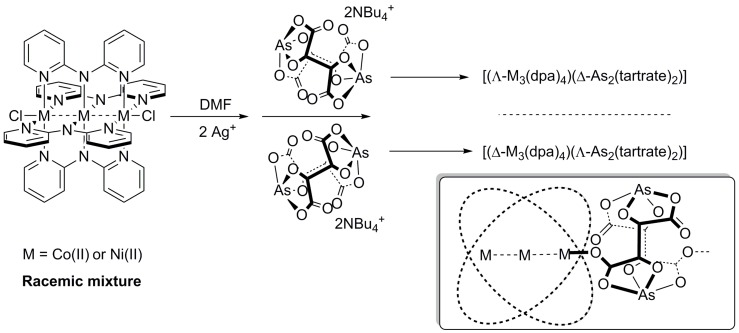
Synthetic approach employed to prepare chiral one dimensional Coordination Polymers (CPs) of the general formula [(M_3_(dpa)_4_)(As_2_(tartrate)_2_)] (M = Co(II) and Ni(II)). Inset: Representation of the coordination mode of the [As_2_(tartrate)_2_]^2−^ ligands.

In this work, we report a synthetic procedure to obtain one-dimensional chiral coordination polymers built from [M_3_(dpa)_4_]^2+^ (M = Co(II) and Ni(II)) and [As_2_(tartrate)_2_]^2−^ units (Figure 2). The use of enantiopure Δ- or Λ-[As_2_(tartrate)_2_]^2−^ promotes enantiomeric resolution of the helicoidal [M_3_(dpa)_4_]^2+^ cations by chiral recognition during the self-assembly process, uniquely affording polymers based on [(Δ-Co_3_(dpa)_4_)(Λ-As_2_(tartrate)_2_)]·3DMF (Δ-**1**), [(Λ-Co_3_(dpa)_4_)(Δ-As_2_(tartrate)_2_)]·3DMF (Λ-**1**), [(Δ-Ni_3_(dpa)_4_)(Λ-As_2_(tartrate)_2_)]·(4 − *n*)DMF∙*n*Et_2_O (Δ-**2**) or [(Λ-Ni_3_(dpa)_4_)(Δ-As_2_(tartrate)_2_)]·(4 − *n*)DMF∙*n*Et_2_O (Λ-**2**) repeating units. ECD measurements in DMF solutions of the two pairs of enantiomers demonstrate the efficiency of the chiral resolution strategy employed and the high stability towards racemization that these enantiopure helices display in solution.

## 2. Experimental Section

### 2.1. Materials

Syntheses were carried out under inert atmosphere using standard glovebox or Schlenk techniques. Acetonitrile (MeCN) and diethyl ether (Et_2_O) were purified using an Inert solvent purification system (Amesbury, MA, USA). Anhydrous *N*,*N*-dimethylformamide (DMF) was purchased from Acros Organics (Geel, Belgium) and used as received, and AgPF_6_ and AgBF_4_ were purchased from Strem (Newburyport, MA, USA) and stored in a nitrogen glovebox. [Co_3_(dpa)_4_Cl_2_] [31], [Ni_3_(dpa)_4_Cl_2_] [32,33,34], and (NBu_4_)_2_[As_2_(tartrate)_2_] (Δ and Λ) [35,36] were prepared as reported elsewhere.

### 2.2. Physical Measurements

CHN elemental analyses were performed by the Service d’Analyse Elémentaire, UMR 7565, Université de Lorraine (Vandoeuvre-lès-Nancy, France). IR spectra were measured in the 4000–550 cm^−1^ range using a Nicolet 6700 FT-IR spectrometer (Waltham, MA, USA) equipped with a SMART-iTR^TM^ accessory. Circular dichroism measurements were performed at 20 °C using a Jasco J815 circular dichroism spectropolarimeter (Tokyo, Japan). The measurements were made using DMF solutions of 1.89 × 10^−5^ M (Δ-**1**), 4.63 × 10^−6^ M (Λ-**1**), 1.59 × 10^−5^ M (Δ-**2**), and 1.86 × 10^−5^ M (Λ-**2**). Class A volumetric flasks and a Mettler MX5 microbalance (Greifensee, Switzerland) with an estimated error of 2 μg/mg were employed in the sample preparation. The spectra were measured with a 2 nm bandwidth and a scan speed of 50 nm/min. The millidegree data were converted to Δ*ε* using the following equation:

$$\Delta \varepsilon =\frac{\theta }{32,980\times c\times l}$$

where *θ* is the dichroic signal measured in millidegrees, *c* is the concentration in M and *l* is the pathlength of the cuvette in cm.

### 2.3. Crystallography

Powder X-ray diffraction measurements were performed using both a PANalytical X’Pert PRO MPD diffractometer (Almelo, The Netherlands) with Bragg-Brentano geometry, Cu-Kα radiation (λ = 1.54184 Å) and a graphite back scattering monochromator or a laboratory-built experimental set-up equipped with a Rigaku Nanoviewer (XRF microsource generator, MicroMax 007HF, Tokyo, Japan), with a 1200-W rotating anode coupled to a confocal Max-FluxH Osmic mirror (Applied Rigaku Technologies, Tokyo, Japan) and a MAR345 image plate detector (MARResearch, Norderstedt, Germany).

Crystals suitable for single crystal X-ray diffraction were selected under immersion oil and were attached to a MiTeGen microloop (Ithaca, NY, USA) in ambient conditions. The crystals were mounted in a stream of nitrogen and centered in the beam using a video camera. A Bruker APEX II Quasar diffractometer (Billerica, MA, USA) with Mo Kα (λ = 0.71073 Å) radiation (Δ-**1**, Λ-**1,** Δ-**2** and [(Λ-Co_3_(dpa)_4_)(Δ-As_2_(tartrate)_2_)] (4-*n*)DMF∙*n*Et_2_O (Λ-**3**)) or a Rigaku FRX diffractometer (Tokyo, Japan) with Cu Kα (λ = 1.54184 Å) radiation (Λ-**2**) were employed. The structures were solved using direct methods [37,38] and refined by least-squares refinement on *F^2^* followed by difference Fourier synthesis [39]. The hydrogen atoms were introduced at idealized positions and were allowed to ride on the neighboring atoms with relative isotropic displacement coefficients. CCDC 1824503-1824507 contain the crystallographic data for this paper. These data can be obtained free of charge from the Cambridge Crystallographic Data Centre via www.ccdc.cam.ac.uk/data_request/cif. Crystal and refinement data are shown in Table 1.

### 2.4. Synthesis

#### 2.4.1. Synthesis of [(Co_3_(dpa)_4_)(As_2_(tartrate)_2_)]·3DMF (Δ-**1**) and (Λ-**1**)

A mixture of [Co_3_(dpa)_4_Cl_2_] (0.15 g, 0.16 mmol), AgBF_4_ (0.07 g, 0.36 mmol) and 3 mL of DMF was stirred at room temperature for 12 h. The resulting deep green suspension was filtered using a VWR syringe filter (0.2 μm, Radnor, USA), added to the corresponding (NBu_4_)_2_[As_2_(tartrate)_2_] (0.30 g, 0.32 mmol), and stirred for 2 h. The solution was filtered and small dark green plates suitable for single crystal X-ray diffraction were obtained after three days by Et_2_O vapor diffusion into the DMF solution. The crystals were kept in the mother liquor in inert atmosphere conditions.

Δ-**1**: Yield: 0.074 g (30%). Anal. Calcd for Co_3_N_12_C_48_H_36_As_2_O_12_·3C_3_H_7_NO (1518.82 g·mol^−1^) C, 45.08; H, 3.78; N, 13.83%. Found: C, 44.82; H, 3.85; N, 13.56%. FT-IR (cm^−1^): 3108(w), 3072(w), 3038(w), 2922(w), 2884(w), 1661(vs), 1606(vs), 1592(vs), 1547(m), 1470(vs), 1455(vs), 1422(vs), 1382(s), 1367(s), 1337(m), 1314(m), 1263(m), 1155(m), 1127(m), 1091(m), 1076(m), 1061(m), 1021(m), 982(w), 924(w), 898(m), 760(s), 733(s), 658(s), 634(s), 572(m), 561(m).

Λ-**1**: Yield: 0.083 g (34%). Anal. Calcd for Co_3_N_12_C_48_H_36_As_2_O_12_·3C_3_H_7_NO (1518.82 g·mol^−1^) C, 45.08; H, 3.78; N, 13.83%: C, 44.88; H, 3.94; N, 13.42%. FT–IR (cm^−1^): 3111(w), 3074(w), 3038(w), 2920(w), 2884(w), 1663(vs), 1606(vs), 1592(vs), 1547(m), 1470(vs), 1455(vs), 1422(vs), 1382(s), 1366(s), 1337(m), 1314(m), 1263(m), 1155(m), 1127(m), 1091(m), 1075(m), 1060(m), 1021(m), 982(w), 924(w), 898(m), 760(s), 734(s), 659(s), 634(s), 573(m), 561(m).

#### 2.4.2. Synthesis of [Ni_3_(dpa)_4_[As_2_(tartrate)_2_]·DMF (Δ-**2**) and (Λ-**2**)

A solution of [Ni_3_(dpa)_4_Cl_2_] (0.15 g, 0.16 mmol) in 3 mL of DMF and a solution of AgPF_6_ (0.08 g, 0.32 mmol) in 1 mL of DMF were combined and stirred overnight. The resulting suspension was filtered using a VWR syringe filter (0.2 μm, Radnor, USA) and added to the corresponding (NBu_4_)_2_[As_2_(tartrate)_2_] (0.30 g, 0.32 mmol). The solution was stirred for 2 h, filtered using a VWR syringe filter (0.2 μm, Radnor, PA, USA) and dark purple crystals that are suitable for single crystal X-ray diffraction were obtained after several days by Et_2_O vapor diffusion into the DMF solution. The crystals were washed with diethyl ether and dried in the air.

Δ-**2**: Yield: 0.068 g (31%). Anal. Calcd for Ni_3_C_48_H_36_N_12_As_2_O_12_·C_3_H_7_NO·3H_2_O (1425.95 g·mol^−1^): C, 42.96; H, 3.46; N, 12.77%. Found: C, 43.22; H, 3.23; N, 12.44%. FT-IR (cm^−1^): 3069 (w), 3028 (w), 2958 (w), 2931 (w), 2881 (w), 1652 (m), 1602 (s), 1591 (s), 1549 (m), 1467 (s), 1459 (s), 1420 (s), 1389 (m), 1351 (s), 1312 (s), 1281 (m), 1264 (m), 1250 (m), 1154 (m), 1130 (m), 1097 (m), 1076 (m), 1056 (m), 1016 (m), 926 (m), 898 (m), 842 (m), 812 (m), 763 (s), 733 (s), 660 (m), 630 (s), 597 (m), 573 (m).

Λ-**2**: Yield: 0.067 g (31%). Anal. Calcd for Ni_3_C_48_H_36_N_12_As_2_O_12_·C_3_H_7_NO·3H_2_O (1425.95 g·mol^−1^): C, 42.96; H, 3.46; N, 12.77%. Found: C, 42.92; H, 3.29; N, 12.30%. FT-IR (cm^−^^1^): 3073 (w), 3028 (w), 2972 (w), 2931 (w), 2889 (w), 1644 (m), 1603 (s), 1591(s), 1549 (m), 1468 (s), 1459 (s), 1420 (s), 1352 (s), 1312 (s), 1283 (m), 1264 (m), 1250 (m), 1153 (m), 1125 (m), 1073 (m), 1056 (m), 1016 (m), 929 (m), 897 (m), 841 (m), 811 (m), 763 (s), 734 (s), 661 (m), 632 (s), 597 (m), 574 (m).

## 3. Results and Discussion

### 3.1. Synthesis

In this work, a similar approach to that recently employed to obtain the coordination polymers [Co_3_(dpa)_4_MF_6_]·2DMF (M = Zr(IV), Sn(IV), Re(IV), Ir(IV), Os(IV)) has been used [20,21]. The protocol consists of two synthetic steps, first removing the axial chloride ligands in [M_3_(dpa)_4_Cl_2_] with a silver salt, and then replacing the counteranion by a polytopic ligand, in this case, arsenyl tartrate, thus allowing for the construction of polymeric structures. When this reaction was performed in acetonitrile, the chiral resolution of the [Co_3_(dpa)_4_(MeCN)_2_]^2+^ clusters with Δ- or Λ-[As_2_(tartrate)_2_]^2−^ yielded enantiopure zero-dimensional compounds of the general formula [Co_3_(dpa)_4_(MeCN)_2_](NBu_4_)_2_[As_2_(tartrate)_2_]_2_, in which acetonitrile caps the axial position of the trinuclear cluster and [As_2_(tartrate)_2_]^2−^ acts as a counteranion [29]. However, when DMF was used in the anion exchange reaction with Δ- or Λ-[As_2_(tartrate)_2_]^2+^ , enantiomeric resolution was accomplished by the formation of one-dimensional CPs, where axial [As_2_(tartrate)_2_]^2−^ anions connect the clusters, giving [(Δ-Co_3_(dpa)_4_)(Λ-As_2_(tartrate)_2_)]·3DMF (Δ-**1**) or [(Λ-Co_3_(dpa)_4_)(Δ-As_2_(tartrate)_2_)]·3DMF (Λ-**1**), depending on the chirality of the arsenyl tartrate used. A similar procedure was followed with the {Ni_3_} clusters, yielding [(Δ-Ni_3_(dpa)_4_)(Λ-As_2_(tartrate)_2_)]·(4 − *n*)DMF∙*n*Et_2_O (Δ-**2**) or [(Λ-Ni_3_(dpa)_4_)(Δ-As_2_(tartrate)_2_)]·(4 − *n*)DMF∙*n*Et_2_O (Λ-**2**).

Two equivalents of (NBu_4_)_2_[As_2_(tartrate)_2_] were routinely employed in these reactions, giving a crystalline yield of ca. 30% in all cases. The maximum expected yield is 50%, given that the clusters are not expected to interconvert in solution [25,26,29,30]. The use of 0.5 or 1 equivalent of (NBu_4_)_2_[As_2_(tartrate)_2_] also gave the same compounds, but the yields were considerably lower. (We should point out that 0.5 equivalent of (NBu_4_)_2_[Δ-As_2_(tartrate)_2_] or (NBu_4_)_2_[Λ-As_2_(tartrate)_2_] is the stoichiometric amount that will react with a given enantiomer in 1 equivalent of the racemic mixture of [Co_3_(dpa)_4_]^2+^.) The compounds described in this work could be also obtained by diethyl ether vapor diffusion into DMF solutions of the molecular [Δ-M_3_(dpa)_4_(MeCN)_2_](NBu_4_)_2_[Λ-As_2_(tartrate)_2_]_2_ and [Λ-M_3_(dpa)_4_(MeCN)_2_](NBu_4_)_2_[Δ-As_2_(tartrate)_2_]_2_ species, but in lower yields than in the previously-described direct synthesis.

### 3.2. Crystal Structrures

**Figure 3:**
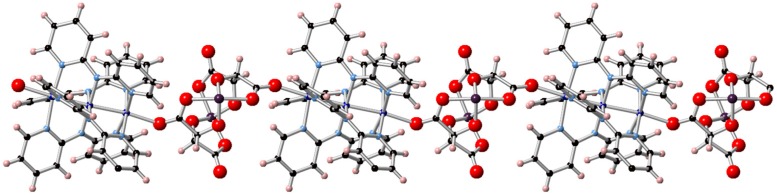
Representation of the coordination polymer in Δ-**1** from X-ray diffraction data. Cobalt: dark blue, nitrogen: light blue, carbon: black, arsenic: purple, oxygen: red, hydrogen: pink.

The crystal structures of Δ-**1** and Λ-**1** were solved and refined in the non*-*centrosymmetric *P*2_1_ space group with final Flack parameters of −0.010(3) and 0.008(9), respectively (Table 1). Bond distances and angles are typical of the starting materials and show no unusual features (Tables S1 and S2). The structures consist of neutral CPs that are formed by alternating [Co_3_(dpa)_4_]^2+^ and [As_2_(tartrate)_2_]^2−^ building blocks. One of the two carboxylate groups of each of the tartrate moieties in the [As_2_(tartrate)_2_]^2−^ units bridges the As and Co ions, giving rise to zig-zag CPs as shown in Figure 3. The cation-anion interactions are heterochiral: [Δ-Co_3_(dpa)_4_]^2+^ helices and [Λ-As_2_(tartrate)_2_]^2−^ form Δ-**1**, whereas [Λ-Co_3_(dpa)_4_]^2+^ helices crystallize exclusively with [Δ-As_2_(tartrate)_2_]^2−^ in Λ-**1**. The {Co_3_} units are symmetrical with respect to the Co−Co distances, 2.306(1) and 2.304(1) Å, in both Δ-**1** and Λ-**1**. To the best of our knowledge, the only other examples of bridging [As_2_(tartrate)_2_]^2^^−^ units are found in [Ag_5_As_4_(C_4_H_2_O_6_)_4_(H_2_O)_5_(X)]_n_ (X = NO_3_^−^, ClO_4_^−^), [Na_8_As_10_(C_4_H_2_O_6_)_8_(C_4_H_3_O_6_)_2_(H_2_O)_19_]_n_, and [Ag_9_As_10_(C_4_H_2_O_6_)_9_(C_4_H_3_O_6_)(H_4_As_2_O_5_)(H_2_O)_10_] [40,41].

In general, the crystals of **1** that we obtained were quite small and weakly diffracting. While the polymer could be directly modeled from the electron density map, the solvent molecules of crystallization were not always apparent. For example, Δ-**1** and Λ-**1** crystallize with a total of three DMF molecules. In Δ-**1**, the crystal data was of sufficient quality to model all three DMF molecules anisotropically. However, in Λ-**1**, only one of the DMF molecules could be satisfactorily modeled, and the presence of the remaining two DMF molecules was confirmed using the PLATON SQUEEZE procedure [42]. Elemental and thermogravimetric (Figure S1) analyses on Δ-**1** and Λ-**1** furthermore confirm a total of three DMF molecules per formula unit in the crystal structure.

Powder X-ray diffraction measurements (PXRD) were performed on samples that were obtained both by direct synthesis in DMF and by recrystallization of [Co_3_(dpa)_4_(MeCN)_2_](NBu_4_)_2_[As_2_(tartrate)_2_]_2_ complexes from DMF/Et_2_O. Comparison of the diffractograms with those simulated from the single-crystal diffraction experiments, suggested that the samples consist of only one phase (Figures S2 and S3). However, a second phase of the coordination polymer, Λ-**3**, crystallizing in the non*-*centrosymmetric *C*2 space group was discovered in a single crystal obtained by recrystallization of [Λ-Co_3_(dpa)_4_(MeCN)_2_](NBu_4_)_2_[Δ-As_2_(tartrate)_2_]_2_ from a dilute (1 mg/mL) DMF solution (Λ-**3,** Table 1 and Table S3).

Surprisingly, the nickel-based compounds Δ-**2** and Λ-**2** were found to be isostructural with this latter structure. While crystals of **2** also proved to be quite small and weakly diffracting, the structures could be solved and refined in the *C*2 space group with Flack parameters of 0.037(5) and 0.017(15) (Table 1). The asymmetric unit in Δ-**2** and Λ-**2** consists of two {Ni_3_}^2+^ units and two [As_2_(tartrate)_2_]^2−^ units. Five DMF molecules per asymmetric unit could be visualized from the electron density map, but remaining Q-peaks suggested the presence of additional disordered solvent. The PLATON SQUEEZE procedure performed on a model retaining two of the interstitial DMF molecules gave results corresponding to approximately six additional DMF and/or diethyl ether molecules per asymmetric unit for a total of four solvent molecules per [(Ni_3_(dpa)_4_)(As_2_(tartrate)_2_)] formula unit. The Ni−Ni distances in Δ-**2** and Λ-**2** are in the 2.389(1)–2.406(1) Å range, which are typical values for linear nickel clusters that do not display M−M bonding, and other bond distances and angles showed no unusual features (Tables S4 and S5). Single-phase samples were obtained, as shown by PXRD measurements (Figures S4 and S5).

**Figure 4:**
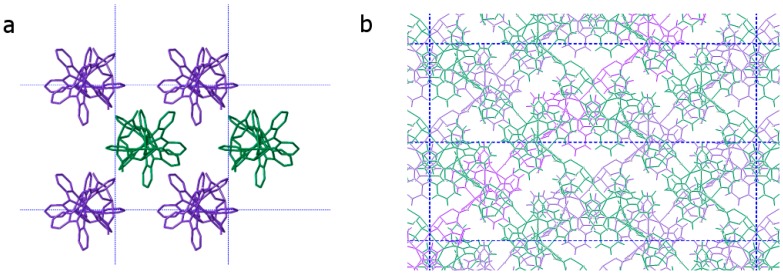
(**a**) Representation of (**a**) Δ-**1** along the crystal *c* axis, *(***b**) Λ-**2** along the crystal *c* axis. Green and violet polymers are related to each other by a two-fold screw (**a**) or rotation (**b**) axis.

From a molecular point of view, Δ-**2** and Λ-**2** are analogous to Δ-**1** and Λ-**1**, having the same connectivity, coordination modes, and heterochiral interactions between the cations and anions. However, the packing in the crystal is quite different for the two compounds. The cobalt-based chains in the crystal structure of the *P*2_1_ phase propagate along the *c* crystal axis and are thus globally parallel to one another, orienting themselves in an approximation of cubic close packing (Figure 4a). On the other hand, the nickel-based chains, crystallizing in the *C*2 phase, are not aligned with any cell axis, but rather form sheets of polymers in the *ab* plane. The polymers within a sheet are parallel to one another, and each sheet is oriented such that the polymers are orthogonal to those of an adjacent sheet (Figure 4b). Indeed, the difficulty in modelling the solvent molecules in the *C*2 phase is likely due to this lamellar packing in the three-dimensional structure. Solvent molecules are free to move in the spaces between the sheets, and thermogravimetric analysis (Figure S6) and elemental analysis made on samples that were stored in the air show that the solvents can be easily lost and replaced by adventitious water molecules.

### 3.3. Circular Dichroism Studies

**Figure 5:**
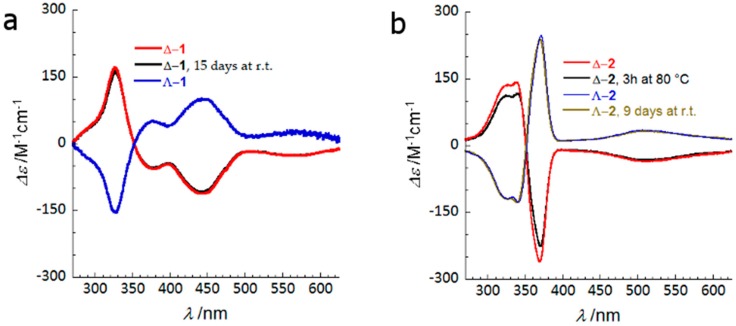
ECD spectra measured at 20 °C for DMF solutions of (**a**) Δ-**1** (red), Λ-**1** (blue) and Δ-**1** after 15 days (black) and (**b**) Δ-**2** (red), Λ-**2** (blue), Λ-**2** after 9 days (brown) and Δ-**2** after being heated 3 h at 80 °C (black).

Electronic circular dichroism (ECD) measurements were performed on DMF solutions of Δ-**1**, Λ-**1**, Δ-**2**, and Λ-**2**. Mirror-image dichroic signals were obtained at 20 °C in the 650–270 nm range. The comparable intensity of these signals, arising from two solutions prepared and measured separately, exclude the fast racemization of the complexes in solution (Figure 5). All of the observed peaks correspond to transitions in the [M_3_(dpa)_4_]^2+^ helices, as the [As_2_(tartrate)_2_]^2−^ peaks appear below 250 nm [35,36] and are thus masked by the strong absorption of the solvent below 270 nm. The study of the arsenyl tartrate peaks is indeed difficult in this type of complex because it has been shown that acetonitrile solutions of chiral [Co_3_(dpa)_4_(MeCN)_2_][PF_6_]_2_ complexes also display strong dichroic signals in the 270–190 nm range when measured in MeCN [29].

The stability towards racemization of the [M_3_(dpa)_4_]^2+^ units in solution was investigated by measuring the CD spectra of DMF solutions of Δ-**1** and Λ-**2** after 15 and 9 days of preparation, respectively. No significant decrease of the dichroic signals was found when compared with those of the freshly prepared solutions (Figure 5). The robustness of these polynuclear helices in solution was also demonstrated by a decrease of only 15% in the intensity of the dichroism spectrum of Δ-**2** after heating to 80 °C (the highest attainable temperature for our experimental setup) for 3 h. This is similar to what was observed for enantiomerically resolved [Co_3_(dpa)_4_(MeCN)_2_](NBu_4_)_2_[As_2_(tartrate)_2_]_2_ and [Co_3_(dpa)_4_(MeCN)_2_][PF_6_]_2_ complexes, which showed conformational stability in MeCN solution, even in the absence of chiral anions [29]. DMF was chosen for the present experiments, as it was one of the few solvents in which the compounds were sufficiently soluble. The integrity of the polymeric structures in DMF solution is most likely concentration-dependent with oligomeric and/or monomeric structures being favored under highly dilute conditions, such as those that were employed in these measurements. This accounts for the similar spectra and stability observed for **1** and the corresponding molecular species [29].

The anisotropy factors, *g*, namely the ratio of the dichroic absorption coefficients to the isotropic absorption coefficients (Δ*ε*/*ε*), observed for the transitions in Δ-**1** are 2.5 × 10^−3^ at 325 nm, −2.8 × 10^−3^ at 375 nm, −2.2 × 10^−2^ at 460 nm, and −9.7 × 10^−3^ at 550 nm, whereas the anisotropy factors found in Δ-**2** are 2.8 × 10^−3^ at 320 nm, 3.0 × 10^−3^ at 340 nm, −1.4 × 10^−2^ at 375 nm, and −1.2 × 10^−2^ at 500 nm. The value of *g* is related to the nature of the transition. Absolute values equal or less than 5 × 10^−3^ are associated with electric dipole allowed-magnetic dipole forbidden transitions such as charge transfer and ligand π−π* transitions, while magnetic dipole allowed-electric dipole forbidden transitions such as ligand field transitions give absolute values equal or higher than 5 × 10^−3^ [43]. Thus, based on the large *g* values, the two lowest-energy transitions in each spectrum likely correspond to metal-centered transitions. The highest Δ*ε*/*ε* value obtained in the present polymers is −2.2 × 10^−2^ for a metal-centered transition at 460 nm in Δ-**1**, which is comparable to that found in the ^1^B_b_ bands of [7] helicene [44].

## 4. Conclusions

Chiral [As_2_(tartrate)_2_]^2^^−^ anions have been successfully employed to resolve racemic mixtures of [M_3_(dpa)_4_]^2+^ (M = Co(II), Ni(II)) cations, while simultaneously acting as bridging units in the self-assembly of chiral coordination polymers. The isolated optically active compounds give strong dichroic signals in the UV-vis range with high anisotropy factors for metal-centered transitions. This chiral resolution method may potentially reveal even higher anisotropy factors when applied to the resolution of linear clusters of higher nuclearity. The chiral [M_3_(dpa)_4_]^2+^ helices are very stable in DMF solution, showing no change in the ECD spectra after several days at room temperature and only a small decrease in the signals after being heated at 80 °C. We are now investigating different types of dichroism in these complexes and exploring the preparation of chiral [M_3_(dpa)_4_]^2+^ with different axial ligands.

## Figures and Tables

**Table 1:** Crystallographic data for complexes Δ-**1**, Λ-**1**, Δ-**2**, Λ-**2,** and Λ-**3**.

Crystallographic parameters	Δ-1	Λ-1	Δ-2 ^a^	Λ-2 ^a^	Λ-3 ^a^
λ/Å	0.71073	0.71073	0.71073	1.54184	0.71073
*T*/K	130(10)	120(2)	120(2)	120(2)	120(2)
Formula	C_57_H_56_As_2_Co_3_ N_15_O_15_	C_57_H_56_As_2_Co_3_ N_15_O_15_	C_102_H_86_As_4_N_26_ Ni_6_O_26_	C_102_H_86_As_4_N_26_ Ni_6_O_26_	C_102_H_86_As_4_Co_6_ N_26_O_26_
fw	1517.79	1517.79	2743.90	2743.90	2745.22
Space group	*P*2_1_	*P*2_1_	*C*2	*C*2	*C*2
*a*/Å	13.5231(5)	13.3130(9)	45.622(5)	45.5728(6)	45.879(3)
*b*/Å	14.7889(5)	14.9023(11)	13.7163(14)	13.6960(2)	13.7591(9)
*c*/Å	15.7030(5)	15.7246(11)	20.815(2)	20.6775(4)	20.3544(14)
*β*/°	98.8470(10)	97.981(3)	94.431(5)	94.589(2)	95.307(4)
*V*/Å^3^	3103.11(18)	3089.5(4)	12,986(2)	12,864.8(4)	12,793.8(14)
*Z*	2	2	4	4	4
*d* calc (g/cm^3^)	1.624	1.632	1.403	1.417	1.425
*μ* (mm^−1^)	1.930	1.938	1.937	2.716	1.861
*R* indices (all data) ^b^	*R*_1_ = 0.0292 *wR*_2_ = 0.0690	*R*_1_ = = 0.0940 *wR*_2_ = 0.1449	*R*_1_ = 0.0896 *wR*_2_ = 0.1396	*R*_1_ = 0.0541 *wR*_2_ = 0.1360	*R*_1_ = 0.0433 *wR*_2_ = 0.0859
GooF on *F*^2^	0.995	1.020	1.020	1.043	1.051
Flack parameter	−0.010(3)	0.008(9)	0.037(5)	0.017(15)	0.010(3)

^a^ Data reported for structure with only two DMF solvents (out of eight total solvents of crystallization) modeled, and therefore formula weight and density are artificially low; ^b^ *R*_1_ = Σ||*F*_o_| − |*F*_c_||/Σ|*F*_o_|; *wR*_2_ = [Σ[*w*(*F*_o_^2^ − *F*_c_^2^)^2^]/Σ[*w*(*F*_o_^2^)^2^]]^1/2^, *w* = 1/σ^2^(*F*_o_^2^) + (*aP*)^2^ + *bP*, where *P* = [max(0 or *F*_o_^2^) + 2(*F*_c_^2^)]/3.

## Supplementary Materials

The following are available online at http://www.mdpi.com/2073-4360/10/3/311/s1: Table S1: Selected bond lengths [Å] for Δ-**1**, Table S2: Selected bond lengths [Å] for Λ-**1**, Figure S1: Thermogravimetric analysis for Δ-**1**, Figure S2: powder diffraction pattern of Λ-**1**, Figure S3: powder diffraction pattern of Δ-**1**, Table S3: Selected bond lengths [Å] for Λ-**3**, Table S4: Selected bond lengths [Å] for Δ-**2**, Table S5: Selected bond lengths [Å] for Λ-**2**. Figure S4: Powder diffraction pattern of Λ-**2**, Figure S5: Powder diffraction pattern of Δ-**2**, Figure S6: Thermogravimetric analysis for Δ-**2**.
