# Supplementary material for: Enantiopure Chiral Coordination Polymers Based on Polynuclear Paddlewheel Helices and Arsenyl Tartrate
Source: Polymers (Basel). 2018 Mar 13;10(3):311. doi: 10.3390/polym10030311 (PMC6414907; doi:10.3390/polym10030311)
Supplement: Supplementary file 1 [file polymers-10-00311-s001.docx]

**Supplementary Materials: Enantiopure Chiral Coordination Polymers based on Polynuclear Paddlewheel Helices and Arsenyl Tartrate**

Ángela Valentín-Pérez, Ahmad Naim, Elizabeth A. Hillard*, Patrick Rosa and Miguel Cortijo*

**Table S1.** Selected bond lengths [Å] for Δ-**1.**

| As1 | O1 | 2.003(3) | Co1 | N4 | 1.975(4) |
| --- | --- | --- | --- | --- | --- |
| As1 | O10 | 1.805(4) | Co2 | N8 | 1.904(4) |
| As1 | O13 | 2.078(3) | Co2 | N11 | 1.907(4) |
| As1 | O3 | 1.784(3) | Co2 | N5 | 1.910(4) |
| As2 | O7 | 2.034(4) | Co2 | N2 | 1.893(4) |
| As2 | O4 | 1.801(3) | Co2 | Co3 | 2.3040(9) |
| As2 | O6 | 1.983(3) | Co3 | O12 | 2.176(3) |
| As2 | O9 | 1.806(3) | Co3 | Co2 | 2.3040(9) |
| Co1 | Co2 | 2.3060(9) | Co3 | N12 | 1.975(4) |
| Co1 | O2 | 2.146(3) | Co3 | N6 | 1.953(4) |
| Co1 | N10 | 1.976(4) | Co3 | N9 | 1.987(4) |
| Co1 | N7 | 1.971(4) | Co3 | N3 | 1.970(4) |
| Co1 | N1 | 1.996(4) | ‐ | ‐ | ‐ |

**Table S2.** Selected bond lengths [Å] for Λ-**1.**

| As1 | O1 | 1.993(6) | Co1 | N7 | 1.974(9) |
| --- | --- | --- | --- | --- | --- |
| As1 | O3 | 1.785(7) | Co1 | N10 | 1.996(8) |
| As1 | O10 | 1.801(8) | Co2 | Co3 | 2.304(8) |
| As1 | O13 | 2.075(7) | Co2 | N2 | 1.89(1) |
| As2 | O4 | 1.810(7) | Co2 | N5 | 1.90(1) |
| As2 | O6 | 1.981(9) | Co2 | N8 | 1.86(1) |
| As2 | O7 | 2.03(1) | Co2 | N11 | 1.93(1) |
| As2 | O9 | 1.799(8) | Co3 | N3 | 1.964(8) |
| Co1 | Co2 | 2.306(9) | Co3 | N6 | 1.955(7) |
| Co1 | O2 | 2.158(7) | Co3 | N9 | 2.004(9) |
| Co1 | N1 | 1.99(1) | Co3 | N12 | 1.958(9) |
| Co1 | N4 | 1.980(9) | ‐ | ‐ | ‐ |

**Figure S1.** Thermogravimetric analysis for Δ**-1**. Heating ramp: 5.00 °C/min to 30 °C, 120 min at 30.00 °C, heating ramp of 5°C/min from 30 °C to 550°C.

**Figure S2.** Comparison of powder diffraction pattern of Λ-**1** obtained by direct synthesis (black) with those simulated from single crystal diffraction data of the *P*2_1_ phase (red) and *C*2 phase (blue) of [(Λ-Co_3_(dpa)_4_)(Δ-As_2_(tartrate)_2_)].

**Figure S3**. Comparison of powder diffraction pattern of Δ-**1** obtained by recrystallization of [Δ-Co_3_(dpa)_4_(MeCN)_2_](NBu_4_)_2_[Λ-As_2_(tartrate)_2_]_2_ in DMF (black) with those simulated from single crystal diffraction data of the *P*2_1_ phase (red) and *C*2 phase (blue) of [(Λ-Co_3_(dpa)_4_)(Δ-As_2_(tartrate)_2_)].

**Table S3.** Selected Bond lengths [Å] Λ-**3**.

| As1 | O1 | 2.043(5) | Co2 | N5 | 1.895(5) |
| --- | --- | --- | --- | --- | --- |
| As1 | O3 | 1.801(4) | Co2 | N8 | 1.917(4) |
| As1 | O7 | 1.790(4) | Co2 | N11 | 1.903(6) |
| As1 | O8 | 1.987(4) | Co3 | O13 | 2.120(4) |
| As2 | O4 | 1.799(4) | Co3 | N3 | 1.981(6) |
| As2 | O6 | 2.020(4) | Co3 | N6 | 1.982(6) |
| As2 | O9 | 1.993(4) | Co3 | N9 | 1.979(6) |
| As2 | O10 | 1.793(4) | Co3 | N12 | 1.968(6) |
| As3 | O16 | 1.796(5) | Co4 | O24 | 2.154(4) |
| As3 | O17 | 2.035(8) | Co4 | N22 | 2.010(4) |
| As3 | O19 | 2.027(8) | Co4 | N13 | 1.989(4) |
| As3 | O21 | 1.812(5) | Co4 | N16 | 1.967(5) |
| As4 | O14 | 2.021(4) | Co4 | N19 | 1.971(4) |
| As4 | O15 | 1.786(4) | Co5 | Co4 | 2.306(1) |
| As4 | O22 | 1.797(4) | Co5 | Co6 | 2.297(1) |
| As4 | O23 | 2.027(4) | Co5 | N14 | 1.890(5) |
| Co2 | Co1 | 2.294(1) | Co5 | N23 | 1.888(4) |
| Co1 | O2 | 2.114(4) | Co5 | N17 | 1.909(5) |
| Co1 | N1 | 1.964(5) | Co5 | N20 | 1.925(6) |
| Co1 | N4 | 1.984(5) | Co6 | N24 | 1.976(4) |
| Co1 | N7 | 1.984(4) | Co6 | N18 | 1.971(4) |
| Co1 | N10 | 1.979(6) | Co6 | N21 | 1.948(6) |
| Co3 | Co2 | 2.295(1) | Co6 | N15 | 2.008(5) |
| Co2 | N2 | 1.892(5) | Co6 | O11 | 2.119(4) |

Table S4. Selected bond lengths [Å] for Δ−2.

| As1 | O1 | 2.059(7) | Ni2 | N8 | 1.93(1) |
| --- | --- | --- | --- | --- | --- |
| As1 | O3 | 1.787(7) | Ni2 | N11 | 1.86(1) |
| As1 | O7 | 1.786(8) | Ni3 | O13 | 2.022(8) |
| As1 | O8 | 2.003(7) | Ni3 | N3 | 2.105(9) |
| As2 | O4 | 1.806(7) | Ni3 | N6 | 2.060(8) |
| As2 | O6 | 2.022(8) | Ni3 | N9 | 2.08(1) |
| As2 | O9 | 1.998(8) | Ni3 | N12 | 2.07(1) |
| As2 | O10 | 1.784(7) | Ni5 | Ni4 | 2.406(2) |
| As3 | O16 | 1.804(8) | Ni4 | O24 | 2.027(7) |
| As3 | O17 | 2.00(1) | Ni4 | N13 | 2.087(8) |
| As3 | O19 | 2.01(1) | Ni4 | N16 | 2.097(8) |
| As3 | O21 | 1.820(8) | Ni4 | N19 | 2.084(8) |
| As4 | O14 | 2.025(7) | Ni4 | N22 | 2.098(8) |
| As4 | O15 | 1.783(8) | Ni6 | Ni5 | 2.400(2) |
| As4 | O22 | 1.788(7) | Ni5 | N14 | 1.908(8) |
| As4 | O23 | 2.057(7) | Ni5 | N17 | 1.874(8) |
| Ni2 | Ni1 | 2.394(2) | Ni5 | N20 | 1.860(9) |
| Ni1 | O2 | 2.014(7) | Ni5 | N23 | 1.91(1) |
| Ni1 | N1 | 2.035(8) | Ni6 | O11 | 2.024(7) |
| Ni1 | N4 | 2.089(8) | Ni6 | N15 | 2.084(8) |
| Ni1 | N7 | 2.108(8) | O11 | Ni6 | 2.024(7) |
| Ni1 | N10 | 2.068(8) | Ni6 | N18 | 2.100(8) |
| Ni3 | Ni2 | 2.397(2) | Ni6 | N21 | 2.06(1) |
| Ni2 | N2 | 1.855(9) | Ni6 | N24 | 2.083(9) |
| Ni2 | N5 | 1.920(9) | ‐ | ‐ | ‐ |

**Table S5.** Selected bond lengths [Å] for Λ−**2**.

| O1 | As1 | 2.072(5) | N8 | Ni2 | 1.914(6) |
| --- | --- | --- | --- | --- | --- |
| O3 | As1 | 1.789(5) | N11 | Ni2 | 1.877(7) |
| O7 | As1 | 1.780(6) | O13 | Ni3 | 2.043(6) |
| O8 | As1 | 2.010(5) | Ni2 | Ni3 | 2.391(2) |
| O4 | As2 | 1.806(6) | N3 | Ni3 | 2.105(6) |
| O6 | As2 | 2.035(6) | N6 | Ni3 | 2.076(6) |
| O9 | As2 | 1.999(6) | N9 | Ni3 | 2.067(6) |
| O10 | As2 | 1.788(5) | N12 | Ni3 | 2.063(7) |
| O16 | As3 | 1.793(6) | Ni4 | Ni5 | 2.397(1) |
| O17 | As3 | 1.97(1) | N13 | Ni4 | 2.072(5) |
| O19 | As3 | 2.03(1) | O24 | Ni4 | 2.042(6) |
| O21 | As3 | 1.803(6) | N16 | Ni4 | 2.087(6) |
| O14 | As4 | 2.019(5) | N19 | Ni4 | 2.084(6) |
| O15 | As4 | 1.790(6) | N22 | Ni4 | 2.099(6) |
| O22 | As4 | 1.779(5) | Ni5 | Ni6 | 2.393(1) |
| O23 | As4 | 2.061(5) | N14 | Ni5 | 1.899(6) |
| Ni1 | Ni2 | 2.389(1) | N17 | Ni5 | 1.892(6) |
| O2 | Ni1 | 2.044(5) | N20 | Ni5 | 1.870(6) |
| N1 | Ni1 | 2.066(6) | N23 | Ni5 | 1.917(6) |
| N4 | Ni1 | 2.100(6) | O11 | Ni6 | 2.046(5) |
| N7 | Ni1 | 2.102(6) | N15 | Ni6 | 2.096(6) |
| N10 | Ni1 | 2.081(6) | N18 | Ni6 | 2.090(6) |
| N2 | Ni2 | 1.879(6) | N21 | Ni6 | 2.058(6) |
| N5 | Ni2 | 1.908(6) | N24 | Ni6 | 2.071(7) |

**Figure S4.** Comparison of powder diffraction pattern of Λ-**2** obtained by direct synthesis (black) with those simulated from single crystal diffraction data of the *P*2_1_ phase (red) and *C*2 phase (blue) of [(Λ-Co_3_(dpa)_4_)(Δ-As_2_(tartrate)_2_)].

**Figure S5.** Comparison of powder diffraction pattern of Δ-**2** obtained by recrystallization of [Δ-Ni_3_(dpa)_4_(MeCN)_2_](NBu_4_)_2_[Λ-As_2_(tartrate)_2_]_2_ in DMF (black) with those simulated from single crystal diffraction data of the *P*2_1_ phase (red) and *C*2 phase (blue) of [(Λ-Co_3_(dpa)_4_)(Δ-As_2_(tartrate)_2_)].

**Figure S6.** Thermogravimetric analysis for [(Λ-Ni_3_(dpa)_4_)(Δ -As_2_(tartrate)_2_)]·0.5DMF·0.5H_2_O. Heating ramp: 3.00 °C/min to 550 °C.
